# Nitrogen-doped amorphous carbon-silicon core-shell structures for high-power supercapacitor electrodes

**DOI:** 10.1038/srep42425

**Published:** 2017-02-10

**Authors:** S. A. Safiabadi Tali, S. Soleimani-Amiri, Z. Sanaee, S. Mohajerzadeh

**Affiliations:** 1 Nanofabricated Energy Devices Laboratory, Department of Electrical and Computer Engineering, University of Tehran, Tehran, Iran; 2 Thin film and Nanoelectronics Laboratory, Department of Electrical and Computer Engineering, University of Tehran, Tehran, Iran; 3 Department of Electrical and Computer Engineering, Babol Noshirvani University of Technology, Babol, Iran

## Abstract

We report successful deposition of nitrogen-doped amorphous carbon films to realize high-power core-shell supercapacitor electrodes. A catalyst-free method is proposed to deposit large-area stable, highly conformal and highly conductive nitrogen-doped amorphous carbon (*a*-C:N) films by means of a direct-current plasma enhanced chemical vapor deposition technique (DC-PECVD). This approach exploits C_2_H_2_ and N_2_ gases as the sources of carbon and nitrogen constituents and can be applied to various micro and nanostructures. Although as-deposited *a*-C:N films have a porous surface, their porosity can be significantly improved through a modification process consisting of Ni-assisted annealing and etching steps. The electrochemical analyses demonstrated the superior performance of the modified *a*-C:N as a supercapacitor active material, where specific capacitance densities as high as 42 F/g and 8.5 mF/cm^2^ (45 F/cm^3^) on silicon microrod arrays were achieved. Furthermore, this supercapacitor electrode showed less than 6% degradation of capacitance over 5000 cycles of a galvanostatic charge-discharge test. It also exhibited a relatively high energy density of 2.3 × 10^3^ Wh/m^3^ (8.3 × 10^6^ J/m^3^) and ultra-high power density of 2.6 × 10^8^ W/m^3^ which is among the highest reported values.

During the last few decades, different types of amorphous carbon (*a*-C) films have attracted a great attention because of their vast range of outstanding features such as excellent mechanical properties, chemical inertness and long-term stability in various media, good biocompatibility, tunable bandgap by adjusting Sp^2^ and Sp^3^ carbon bonding ratio, and photoconductivity^1^,^2^,^3^,^4^,^5^. Having such properties, the *a*-C films have shown great potential in a vast scope of biotechnology and solar cell applications^2^,^6^,^7^. Despite their promising properties, there are some obstacles against the ideal utilization of *a*-C films. For instance, as a material in optoelectronic devices, *a*-C films suffer from lack of control over their electrical and optical charactersitics^8^. Moreover, in energy-storage devices, they do not seem to be as efficient as certain carbon-based structures e.g. graphene and carbon nanotubes^9^,^10^. To circumvent these shortcomings, it is necessary to modify the electrochemical properties of *a*-C films in order to enhance their efficiency and applicability for energy storage devices.

It has been shown that *a*-C behaves as a weak p-type semiconducting material, which is able to accommodate dopants. Doping of *a*-C with n-type dopants such as phosphorus (P) and nitrogen (N) has been attempted by several researchers^4^,^11^,^12^. Incorporation of N into *a*-C films has been shown to reduce the electrical resistivity, optical bandgap, and defect density of these films. Furthermore, it has been reported that such incorporation minimizes the compressive stress of *a*-C films^13^,^14^. Owing to its gas phase, nitrogen incorporation in *a*-C films is highly controllable, which in turn leads to desired doping concentrations. These favorable properties allow *a*-C:N films to have great potentials in various applications as optoelectronic devices, solar cells, semiconductor devices, and field emission cathodes^4^,^15^,^16^,^17^.

*a*-C films are synthesized using several techniques including pulsed laser deposition, sputtering, ion beam deposition and filtered cathodic vacuum arc method^18^,^19^,^20^,^21^. However, these techniques are generally expensive and require sophisticated reactors and complex steps. Compared to these methods, chemical vapor deposition (CVD) is a simple, low-cost and easily controllable technique and it is commonly used in the fabrication of different types of carbon-based films^22^,^23^,^24^,^25^. In this work, we report a novel and catalyst-free approach to realize large-area, stable, highly conformal and highly-conductive *a*-C:N films by direct-current plasma enhanced chemical vapor deposition technique (DC-PECVD). Although the deposited *a*-C:N films are inherently porous, we have demonstrated that their surface properties could be further improved by nickel-assisted annealing and a subsequent etching step. For the first time, by means of electrochemical analysis of the deposited films, we have shown the promising performance of the nitrogen-doped *a*-C films as a MEMS-compatible supercapacitor electrode. Various methods have been exploited to study the electrical, physical and electrochemical properties of the *a*-C:N films formed on both planar and non-planar substrates.

## Results

### Material characterization

**Figure 1:**
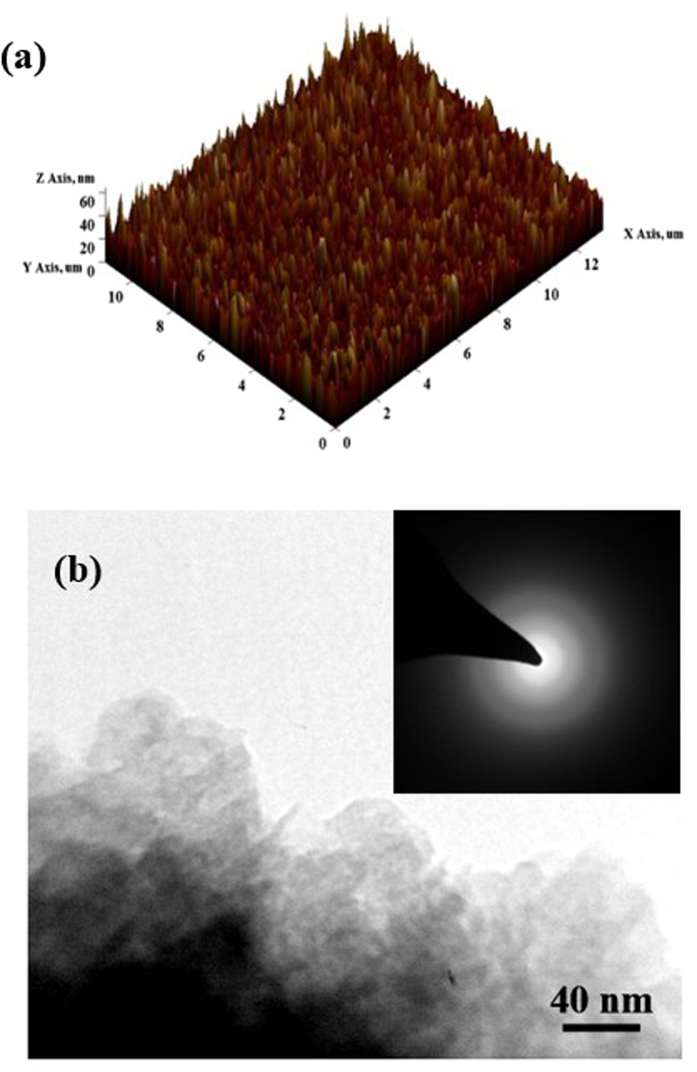
(**a**) Three-dimensional AFM image of the *a*-C:N surface (**b**) cross-sectional TEM image of the *a*-C:N film. SAED pattern of the *a*-C:N layer is depicted in the inset.

Nitrogen-doped amorphous carbon (*a*-C:N) films were deposited by a DC-PECVD method. Physical and chemical properties of the deposited films were identified using scanning electron microscopy (SEM), transmission electron microscopy (TEM), atomic force microscopy (AFM), Raman spectroscopy and X-ray photoelectron spectroscopy (XPS). Figure 1(a) shows an AFM image of the *a*-C:N film deposited on a planar silicon substrate, which demonstrates a rough and porous surface of the layer with a root mean squared (RMS) roughness of about 8 nm. The surface roughness is also evident from the cross-sectional TEM image of *a*-C:N surface as seen in part (b) of this figure. Inset of this image depicts the selected area electron diffraction (SAED) pattern of the *a*-C:N film, indicating its amorphous nature.

**Figure 2:**
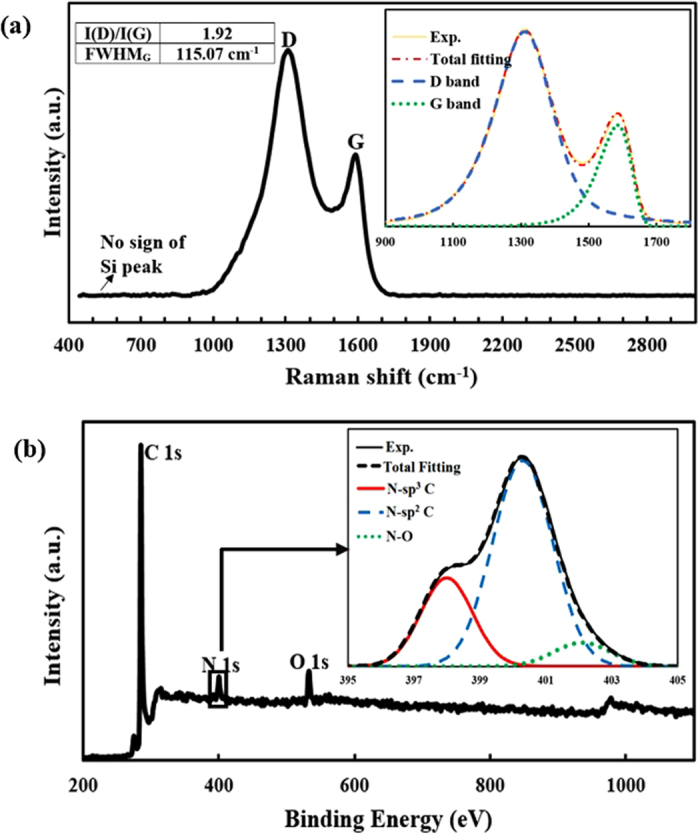
(**a**) The Raman spectra of the *a*-C:N layer (**b**) The XPS spectra of the *a*-C:N layer deposited on Si substrate. The inset shows a representative deconvolution around nitrogen peak.

The Raman spectrum of the deposited *a*-C:N layer is shown in Fig. 2(a). The “G” peak with broad FWHM of 115 cm^−1^ is related to the amorphous nature of *a*-C:N film. Also, the film high I(D)/I(G) ratio (≈1.9) is associated with its appreciable sp^2^ fraction which is an indication of its low band gap^3^,^26^,^27^. In addition, no discernible Raman peak pertaining to the silicon substrate is observed in this figure, demonstrating that the *a*-C:N film has a continuous coverage. The chemical elemental analysis of the *a*-C:N layer on Si substrate was determined by XPS analysis as displayed in Fig. 2(b). The XPS results reveal that the amorphous carbon film has been successfully doped with nitrogen. The presence of oxygen (O) atoms at the surface of the layer is speculated to be due to the oxidation in the air during the sample transferring and handling. Through deconvolution of the XPS spectra, it is deduced that the N atoms in the layer are mostly sp^2^ bonded.

### Evolution of three-dimensional *a*-C:N layers

**Figure 3:**
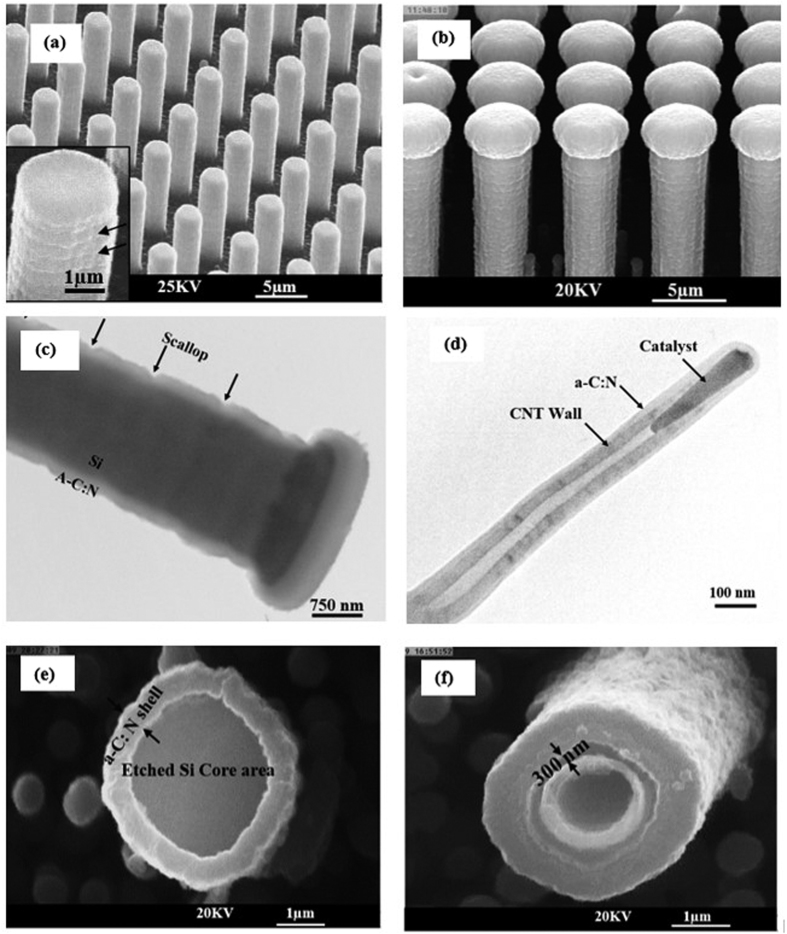
(**a**) SEM image of initial i microrods. The inset shows the scallops on the walls of a microrod (**b**) SEM image of *a*-C:N/Si core-shell microrods (**c**) TEM image of an *a*-C:N/Si core-shell microrod. It is observed that the deposited layer has followed the scallops of the bare microrod (**d**) TEM image of an *a*-C:N/CNT core-shell nanostructure. Finally, (**e**) and (**f**) show the SEM images of a simple and coaxial *a*-C:N microtubes.

To achieve higher surface areas, *a*-C:N films can be realized on three-dimensional structures. Part (a) of Fig. 3 depicts a silicon microrod array fabricated by optical lithography followed by deep reactive ion etching (DRIE)^28^. Figure 3(b) depicts the SEM image of a core-shell microrod array fabricated by coating the silicon microrods with *a*-C:N film. This image reveals that despite the considerable thickness of *a*-C:N film (800 nm), the layer has followed the scallops on the walls of the initial microrods in a conformal fashion. The evolution of scallops is an inevitable result of sequential etching in DRIE processes^29^ as shown in the inset of Fig. 3(a). Parts (c) and (d) of this figure display corresponding TEM images of a microrod and a DC-PECVD grown CNT^30^ coated with *a*-C:N layer with lower thicknesses using a similar technique. These experiments corroborate that in addition to being compatible with MEMS technology, micro/nanostructures of non-silicon materials can also be used as the core for the formation of three-dimensional core-shell features with a controllable *a*-C:N shell thickness. It is also demonstrated that as well as being highly conformal and continuous (even in low-thickness depositions), the technique significantly preserves the surface geometrical features of the core section. The fabricated core-shell micro/nano structures could be further employed as a basis to create high surface area structures. This can be illustrated in Fig. 3(e) and (f) which show free-standing simple and coaxial mictotubes based on *a*-C:N/Si core shell structures. In order to arrive at such microstructures, we have removed the silicon core material from *a*-C:N/Si microrods and *a*-C:N/Si microneedle core-shell structures by using SF_6_ plasma in an RIE unit.

**Figure 4:**
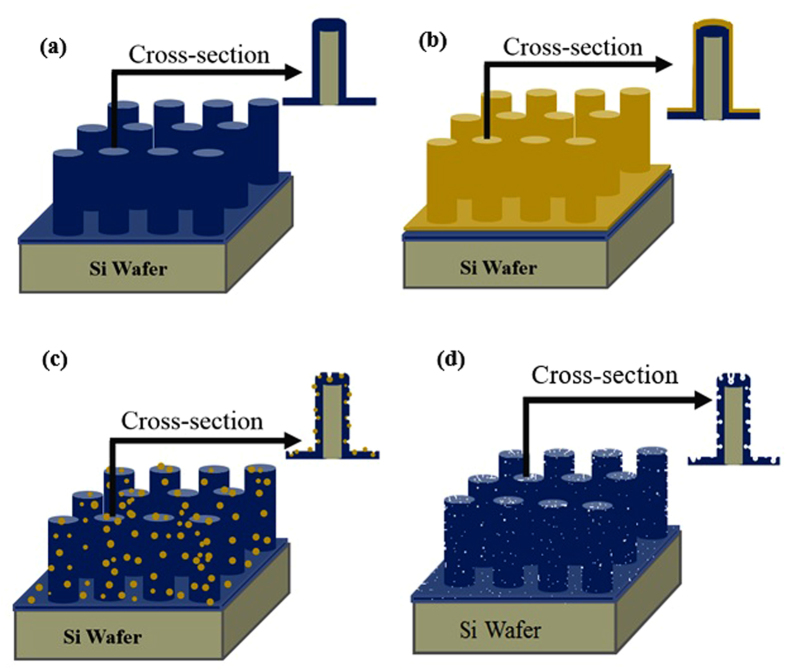
The schematic representation of (**a**) *a*-C:N/Si core-shell microrods array (**b**) Ni thin film deposited on the *a*-C:N/Si core-shell array (**c**) nickel nanoparticles formed by the Ni thin film dewetting and their diffusion into the *a*-C:N film (**d**) *a*-C:N film after etching of nickel nanoparticles.

Although using three-dimensional features as the deposition core is suitable to enhance the real surface area, another further improvement is essential to achieve ultra-high capacity electrochemical devices. In Fig. 4, we have demonstrated the schematic steps of a modification process to enhance the porosity of the as-deposited (pristine) *a*-C:N films. After fabrication of the silicon micro-rod array, it is coated with *a*-C:N layer as seen in part (a) of this figure. This step is then followed by coating a 10 nm nickel film using an electron beam evaporation (part (b)) and annealing at around 1000 °C in a nitrogen ambient for 5 hours (part (c)). During this annealing process, the nickel thin film dewets and changes into nanometer-size nanoparticles. These nickel nanoparticles act as catalysts and a micrographitization process occurs at the *a*-C:N surface^31^,^32^. This graphitization is accompanied by formation of a plethora of nanopores at the *a*-C:N surface, and thus, the real surface area of the film significantly enhances (part (d)).

**Figure 5:**
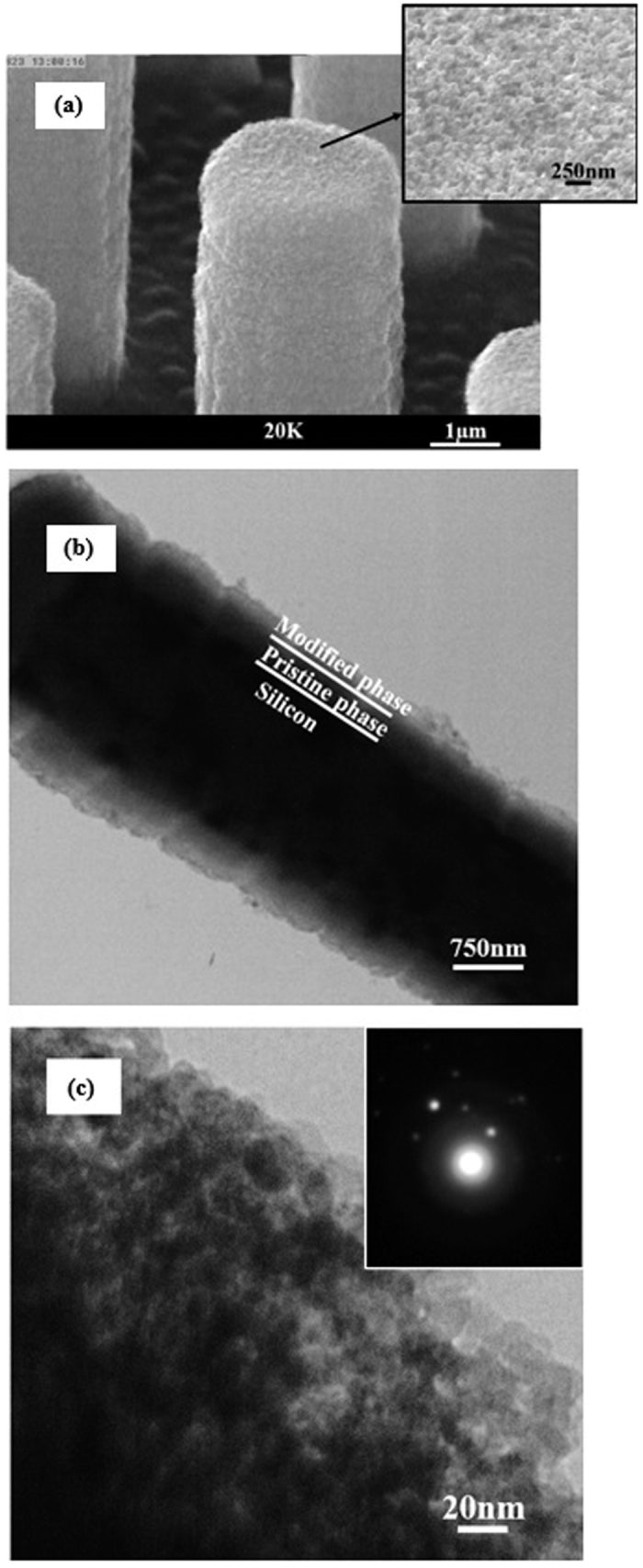
(**a**) SEM image of the *a*-C:N/Si core-shell microrods after Ni deposition and annealing (first modification step). The inset clearly shows porous surface of *a*-C:N after this modification step. (**b**) TEM image of the same sample. The two-phase structure of the *a*-C:N layer is evident. (**c**) A clearer view of the more porous 100 nm thick modified phase. The inset shows a SAED pattern of the modified phase. Dots in this figure could be due to nickel nano-particles.

The evolution of nanopores on the surface of *a*-C:N films is evident from the SEM images of Fig. 5(a) and its inset. Part (b) depicts the TEM image of the same features. It indicates that the thickness of the modified phase is about 100 nm.

Our experiments showed that Ni nanoparticles could be trapped in the *a*-C:N layer pores during the annealing process which could electrochemically react with the electrolyte and thus, hamper the layer stability and its energy performance as a supercapacitor electrode active material. These trapped nanoparticles are evident in the TEM image of the modified phase in Fig. 5(c). Their presence is also characterized by the dots in the modified phase SAED pattern as can be seen in the inset of Fig. 5(c). To eliminate the exposed nickel nanoparticles in the film, the sample was immersed in a nickel etchant solution (HNO_3_-HCl-H_2_O) for about 2 hours. The state of the sample at this stage of modification has been schematically shown in part (d) of Fig. 4.

### Electrochemical characterizations

**Figure 6:**
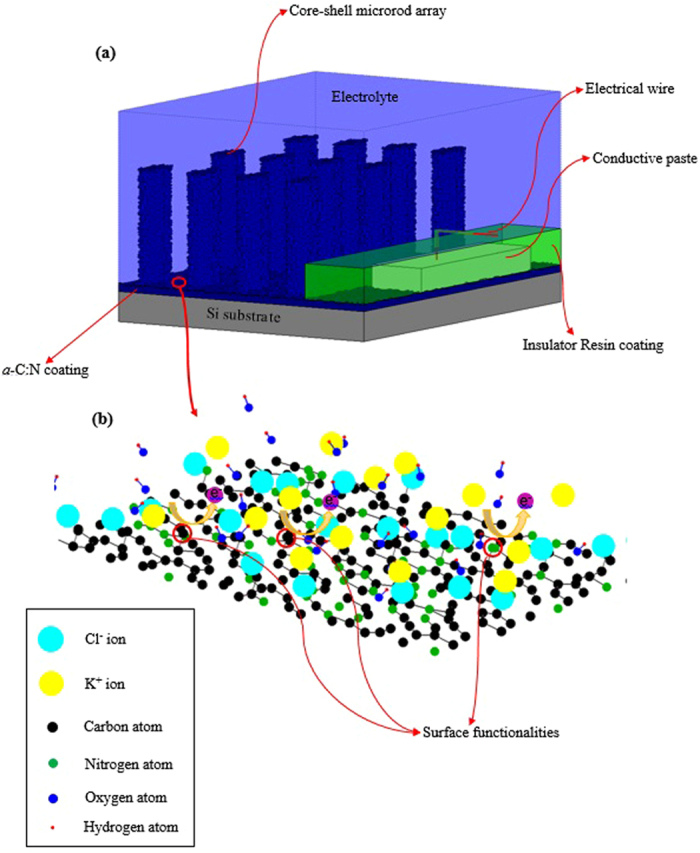
A schematic diagram representing (**a**) the electrode preparation; *a*-C:N coating serves as both the active material and the current collector (**b**) surface functionalities at the pristine *a*-C:N surface clusters nd their faradic reactions in the KCl electrolyte.

In this section, we have studied the electrochemical properties of the supercapacitor electrodes fabricated based on the pristine and modified *a*-C:N coatings on planar and non-planar silicon substrates. Preparation of the samples for the electrochemical analyses is schematically displayed in Fig. 6(a). As shown in this figure, in the assembled electrodes, due to the low resistivity of *a*-C:N coatings, they directly serve as the current collector. This ability immediately gives rise to two major benefits; first, it makes the coatings suitable for on-chip applications. It also enjoys significant flexibility upon choosing the coating substrate for supercapacitor electrodes, such that even non-conductive materials can be utilized. Also, for external contacts of the *a*-C:N, an electrical wire has been attached onto its surface by a conductive paste. As Fig. 6(a) exhibits, in order to isolate the wire and the paste from the electrolyte, they are both coated with an insulator resin coating.

**Figure 7:**
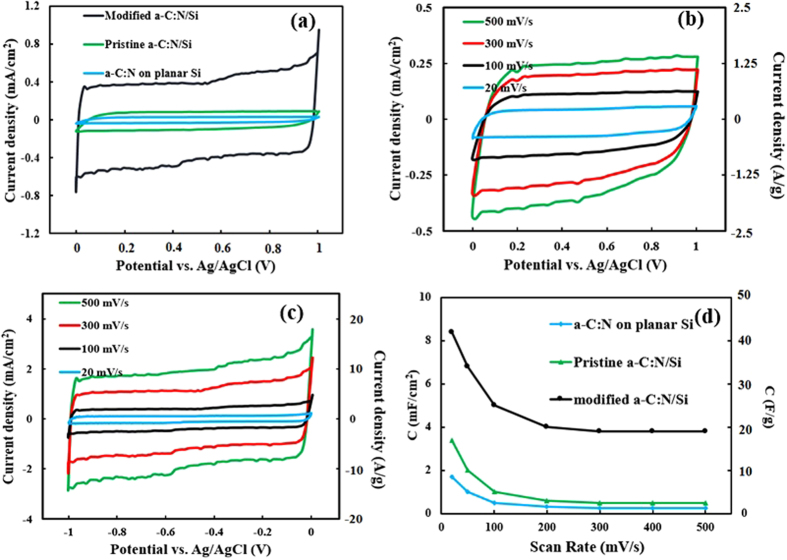
(**a**) cyclic voltammogram (CV) curves of pristine *a*-C:N film on planar Si wafer and Si microrods array, and the modified *a*-C:N/Si core-shell electrode in the potential window of 0 to 1 V and at scan rate of 100 mV/s. (**b**) CV curves of the pristine *a*-C:N/Si core-shell electrode at scan rates of 20 to 500 mV/s. The current densities are reported in areal (mA/cm^2^) and specific (A/g) values. (**c**) CV curves of the modified *a*-C:N/Si core-shell electrode. (**d**) Areal capacitance densities of all the three samples and specific capacitances of the pristine and the modified *a*-C:N films calculated at different scan rates.

Figure 7(a) shows overlaid cyclic voltammogram (CV) curves of the pristine and modified *a*-C:N films on planar Si wafer and Si microrod arrays in a potential window of 0 to 1 V and at scan rate of 100 mV/s. Also, distinct CV curves of the pristine and modified films on Si microrod arrays at scan rates of 20 to 500 mV/s are collected in parts (b) and (c), respectively. Each of the voltammograms was recorded after at least 5 consecutive sequences of cycling when no variation in the form of the curve was observed.

The calculation of specific capacitances of the samples based on CV measurements was done according to the following equation:


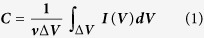


where “C” denotes the capacitance density of the electrodes, “ν” is the scan rate (V/s), “ΔV” is the potential window (V) and “I” is the voltage-dependent discharge current normalized to footprint or mass of the active material (A/cm^2^ or A/g). In the prepared electrodes, the mass of *a*-C:N film as an active material was 0.88 mg. The areal (normalized to footprint) capacitance densities (mF/cm^2^) and specific capacitances (F/g) of the samples at different scan rates are gathered in Fig. 7(d).

The difference between the planar and core-shell pristine *a*-C:N/Si electrodes can be envisaged by observing the results of Fig. 7(a and d). Part (a) exhibits that the core-shell sample produces a larger current density per unit footprint. Correspondingly, as Fig. 7(d) shows, this sample has a larger (nearly two times) areal capacitance density. These larger areal current and capacitance densities are obviously a direct consequence of the effective area enhancement caused by etching the silicon wafer. CV curves of the pristine *a*-C:N/Si core-shell electrode at scan rates of 20 to 500 mV/s are shown in Fig. 7(b). As Fig. 7(b) reveals, the CVs of the pristine *a*-C:N film manifest relatively small Faradic peaks at various scan rates. These peaks are due to charge transfer processes arising from redox reactions of impurities, surface functionalities or unsaturated bonds at the *a*-C:N surface^33^. These surface functionalities are shown in Fig. 6(b) which represents an atomic scale schematic of the interaction between a part of the pristine *a*-C:N surface and the electrolyte.

After the *a*-C:N modification process, the average currents and the corresponding capacitance densities have considerably enhanced (Fig. 7(a,c,d)). The CV curves for the modified core-shell electrode (Fig. 7(c)) resemble rectangles and exhibit no peaks. This implies that no major redox reaction takes place and an efficient double-layer capacitance has been established at the electrode/electrolyte interface for this sample. The CV curves still remain with the same shape even at high scan rates (500 mV/s) with fast transition of the current sign after reversing the scanning direction, indicating a nearly ideal electrochemical capacitive behavior with a relatively low equivalent series resistance (ESR). The observed current increase in the CV curves and the corresponding capacitance density enhancement after the modification (Fig. 7(a,c,d)) imply that the effective surface area of the *a*-C:N film has been enhanced at least four times as a result of the porosity improvement during the modification process. Furthermore, the lower redox rate of this electrode is believed to be due to dissociative distillation or pyrolization of functional groups off the *a*-C:N surface during micrographitization^33^ in the modification process. It is observed for all samples that the highest capacitance corresponds to the lowest scanning rate and with increasing the scan rate, the calculated capacitance decreases to about 50% of the highest value (Fig. 7(d)). This could be related to a transmission-line behavior of the *a*-C:N coatings and their pores^33^.

As explained above, the modified *a*-C:N electrode showed the largest capacitance and lowest redox rate as compared to the other two electrodes. The areal (volumetric) and specific capacitance densities for this electrode were respectively about 8.5 mF/cm^2^ (45 F/cm^3^) and 42 F/g at scan rate of 20 mV/s.

**Figure 8:**
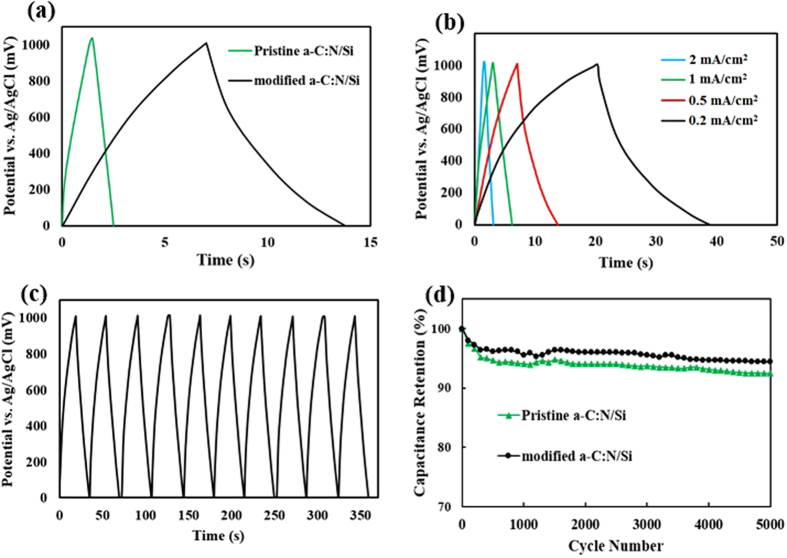
(**a**) Single GCD curves of the pristine and the modified electrodes at 0.5 mA/cm^2^. (**b**) GCD curves of the modified *a*-C:N/Si at different current densities. (**c**) The last ten GCD curves of the modified sample after 5000 cycles at constant current density of 0.2 mA/cm^2^. (**d**) Capacitance retention of the two electrodes over 5000 GCD cycles.

Figure 8(a) compares single galvanostatic charge and discharge (GCD) curves of the pristine and the modified *a*-C:N/Si core-shell electrodes at the same charge–discharge current density of 0.5 mA/cm^2^. This figure reveals that, at the same current density, it takes considerably a larger time (over 5 times) to charge and discharge the modified electrode compared to the pristine one substantiating larger capacitance of the former.

Figure 8(b) represents GCD curves of the modified sample at different current densities ranging from 0.2 to 2 mA/cm^2^. To a good extent, the GCD curves at each current density exhibit a symmetric shape indicating the electrode’s charge–discharge process is highly reversible or, equivalently, it hs a very high columbic efficiency (about 95% on average). Also, no obvious IR drop is observed in either of the curves, which again confirms that the electrode has a low ESR. An identical analysis shows that the pristine electrode has also a low ESR with the average columbic efficiency of about 80%. We evaluated the cycle life of the fabricated electrodes by galvanostatically charging and discharging them for 5000 cycles at the current density of 0.2 mA/cm^2^. The last ten segments of the modified electrode’s successive charge and discharge cycles, shown in Fig. 8(c), indicate an almost stable potential-time response over different cycles. The examination of similar curves corroborate similar stability for the pristine electrode.

The approximate discharge capacitance at each GCD cycle was calculated as below:


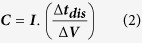


In which “C” is the capacitance of the electrode normalized to unit footprint (F/cm^2^), “I” is the discharge current density (A/cm^2^), “Δt_dis_“ is the discharge time duration, and “ΔV” is the GCD potential window (ΔV = 1 V). Figure 8(d) depicts the calculated capacitance values of the electrodes versus cycle number of continuous GCD measurements. It is observed that the pristine and modified electrodes exhibit 7.5% and 5.6% losses of capacitance during the 5000 cycles, respectively. Preserving above 94% of the initial specific capacitance over 5000 GCD cycles, accompanied by the non-faradic behavior of the modified core-shell electrode (interpreted from its CV and EIS curves) suggests that the modified *a*-C:N electrode is potentially suitable for long-term supercapacitor applications.

**Figure 9:**
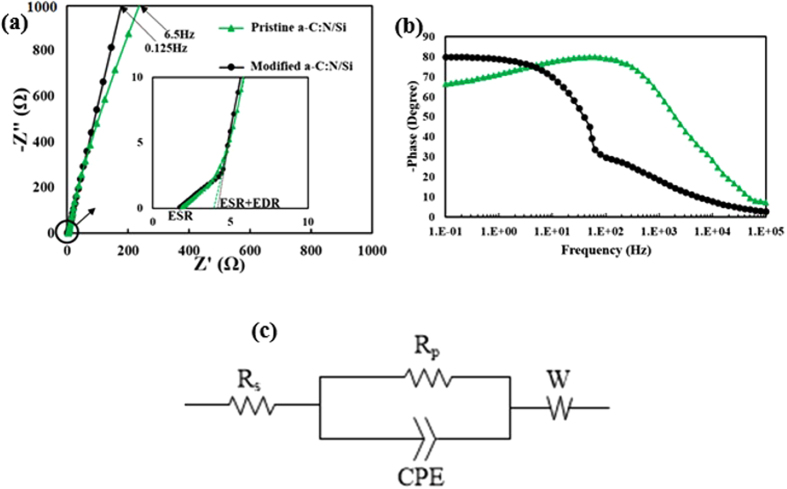
(**a**) Nyquist plots of the two core-shell samples in the range of Z” = 0 to Z” = 1000 Ω. The frequency at which Z” has reached 1000 Ω is labeled for each sample. Inset shows the magnified portion of the Nyquist plots near the origin. (**b**) Impedance phase angles versus frequency (**c**) The equivalent circuit model.

Figure 9(a) shows the Nyquist plots of the two *a*-C:N/Si core-shell samples obtained from EIS measurements. It is seen that the frequency at which the Nyquist lines reach Z” = 1000 Ω has remarkably decreased after the aforementioned *a*-C:N modification. In addition, Z” is inversely proportional to the magnitude of capacitance (since 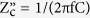), so it is concluded that the capacitance has increased after the modification process. This result is consistent with those obtained from CV and chronopotentiometry measurements.

As part (a) in Fig. 9 depicts, the slope in the mid-frequency region of the Nyquist curve has slightly increased after *a*-C:N modification which is indicative of a decrease in the redox rate^33^, corroborating the results obtained from CV measurements. The inset in Fig. 9(a) shows the shapes of the Nyquist plots at high frequency region. It is seen that while the shape of the Nyquist plot for an ideal capacitor is a vertical line, our samples plots have angles of 45° at the high frequency region. This property arises from the transmission-line behavior of the electrodes^33^.

It is well-known that ESR (Equivalent Series Resistance) can be extracted from Nyquist plot; the intercepts of the plot with Z real axis at high frequency limit of the 45° line corresponds to ESR^33^. As inset of Fig. 9(a) reveals, ESR is about 2 Ω for both samples. The *a*-C:N films act as short-circuit in the high frequency regions; thus, their ESR comprises contact resistance, the electrolyte equivalent series resistance, and the wiring total resistance (ESR = R_wiring_ + R_contact_ + R_electrolyte_).

On the other hand, EDR (Equivalent Distributed Resistance) can also be approximated by the Nyquist plots; as the inset in Fig. 9(a) depicts, extrapolated intercepts on the Z’ axis at infinite frequency is a good measure of ESR + EDR^33^. For both of the electrodes, EDR was about 2 Ω which is due to both the resistance of the *a*-C:N films and the ionic solution resistance within their pores. One main reason for these low values of EDR is the high electrical conductivity of *a*-C:N films. The logarithmic phase angle-frequency diagrams of the electrodes in 0.1 Hz–100 kHz range is depicted in Fig. 9(b). The curves indicate that at lower frequencies, the electrodes show a capacitive behavior with maximum phase angles of about 80°. However, their behavior approaches a purely resistive response at high frequencies. Figure 9(c) shows the corresponding equivalent circuit model related to the impedance spectra of the modified electrode. The fitted model is a parallel combination of a constant phase element (CPE) and a charge transfer resistance (R_p_) in series with an equivalent series resistance (R_s_) and an open circuit generalized finite Warburg (GFW) impedance^34^. As shown in Table 1, the estimated series resistance (R_s_) for the modified electrode is 2 Ω. The constant phase element is defined in terms of its admittance as *Y*_*CEP*_ = *T*_*CEP*_(*jω*)^*n*^ to measure the distributive capacitance of the electrode. The values of T_CPE_ and n for our electrode were estimated to be 1.35 × 10^−2^ and 0.9, respectively. The charge-transfer resistance (R_p_) was extracted as 10 kΩ. This faradic resistance might be a result of charge transfer processes arising from redox reactions of impurities, surface functionalities or unsaturated bonds at the *a*-C:N surface^33^. Finally, to better fit the parallel R-CPE circuit, a reflective Warburg impedance element was used. This Warburg impedance is determined as 
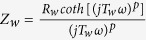
 where, for our sample, *R*_*w*_ = 7 Ω, *T*_*w*_ = 5.2 × 10^−3^ and *p* = 0.45.

**Figure 10:**
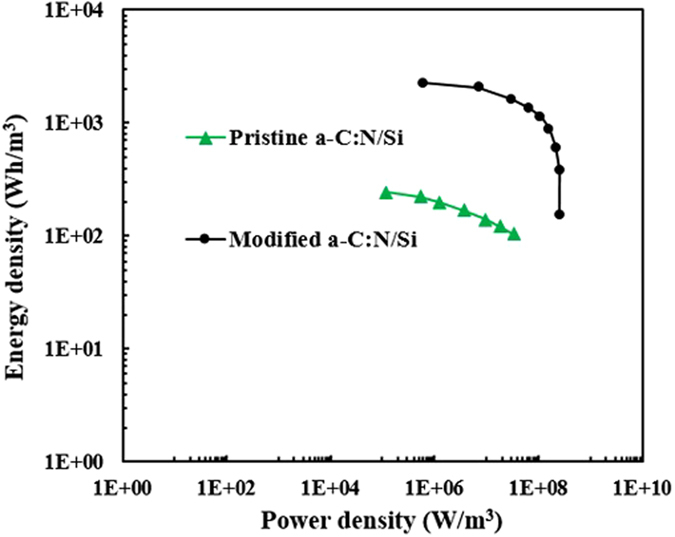
Ragone plots showing volumetric energy and power densities of the pristine and modified *a*-C:N electrodes.

The energy storage devices are further characterized by the amount of power and energy they can deliver to a load^35^. We have extracted the energy (E) and average power density (Pav) based on the CV curves at different rates^36^. The volumetric Ragone plots of the pristine and modified samples are shown in Fig. 10.

The pristine sample yields up to about 2.4 × 10^2^ Wh/m^3^ (8.6 × 10^5^ J/m^3^) below power density of 1.1 × 10^5^ W/m^3^. Moreover, this electrode can deliver the maximum power density of 3.4 × 10^7^ W/m^3^ at the energy density of 1 × 10^2^ Wh/m^3^ (3.6 × 10^5^ J/m^3^). After the *a*-C:N modification, the energy and power density were both significantly enhanced. In a more detailed view, the maximum attainable energy has nearly increased by a factor of 7 and the modified sample delivers up to 2.3 × 10^3^ Wh/m^3^ (8.3 × 10^6^ J/m^3^) below power density of 6 × 10^5^ W/m^3^. Also, the modified sample delivers the maximum power density of 2.6 × 10^8^ W/m^3^ at energy density of 1.5 × 10^2^ Wh/m^3^ (5.4 × 10^5^ J/m^3^). This means that the maximum deliverable power has been enhanced by a factor of about 7.5 and the maximum attainable energy density is improved by a factor of almost 9.5.

The volumetric energy density of the pristine *a*-C:N/Si electrode in our three-electrode system with aqueous electrolyte is not considered a superior value in comparison with some other reported carbon based electrodes which were tested in two-electrode configuration using organic electrolytes^37^,^38^. Aside from the value of its capacitance density, another important reason for this relatively low energy density is the dependence of the energy density on the square of the potential window. The narrow potential window (1 V) dictated by the aqueous electrolyte causes a significant decrease in the energy density of the electrode. However, despite this fact, the maximum volumetric energy density of the modified *a*-C:N/Si electrode is still higher than that of typical activated carbon supercapacitors (<1 mWh/cm^3^)^39^. Interestingly, the modified *a*-C:N/Si electrode manifests a high volumetric power density of 145 W/cm^3^ (even at high energy density around 1 mWh/cm^3^). This volumetric power density is more than 7 times higher than that of conventional supercapacitors^39^,^40^,^41^ and is comparable to that of high power electrolytic capacitors (10^1^–10^3^ W/cm^3^)^42^. The active material’s high conductivity (and the resulting low ESR) is the primary reason for such high powers of the *a*-C:N electrodes which make them good candidates for the fabrication of micro-supercapacitor electrodes.

## Conclusion

In this paper, we have studied the promising application of nitrogen-doped amorphous carbon films as the active material of high-power core-shell supercapacitor electrodes. A catalyst-free approach was proposed for large-area deposition of stable, highly conformal and continuous films of nitrogen-doped amorphous carbon (*a*-C:N) using DC-PECVD. This easy and inexpensive deposition technique has a significant flexibility upon choosing the deposition substrate such that it can coat a variety of both silicon and non-silicon micro/nanostructures with a controllable thickness. The deposited *a*-C:N films have a low resistivity which enables them to directly serve as the current collector when an electrical connection is required. As shown in the manuscript, these qualities make the deposition method suitable for both MEMS and on-chip applications.

Micro-observations of the pristine deposited *a*-C:N films revealed that they have a porous and rough surface. Yet, we much further enhanced their porosity and real surface area by a two-step surface modification process. The first step was Ni-assisted annealing of the films and the second was their exposure to Ni etchant. Then, electrochemical performances of the pristine and the modified *a*-C:N films as supercapacitor electrodes on planar and non-planar silicon substrates were evaluated. Among the evaluated electrodes, the modified *a*-C:N on Si microrod array showed the best performance with a double-layer capacitive behavior. For this sample, the areal, volumetric and specific capacitance densities were, respectively, about 8.5 mF/cm^2^, 45 F/cm^3^ and 42 F/g at scan rate of 20 mV/s and in potential window of 0 to 1 V. Furthermore, it preserved above 94% of its initial specific capacitance value over a 5000-cycle GCD test indicating its potential to be used in long-term supercapacitor applications. Finally, it exhibited the relatively high energy density of 2.3 × 10^3^ Wh/m^3^ (8.3 × 10^6^ J/m^3^) and ultra-high power density of 2.6 × 10^8^ W/m^3^ in an aqueous electrolyte solution. The proposed *a*-C:N deposition method can be further used for fabrication of flexible energy storage/conversion devices and also other functional micro/nanodevices.

## Methods

### *a*-C:N deposition

We employed direct-current plasma-enhanced chemical vapor deposition (DC-PECVD) method for evolution of the amorphous carbon films. First, silicon samples were loaded into a quartz tube furnace and heated up to 850 °C under a nitrogen atmosphere. After stabilization of the temperature in the furnace, the deposition of amorphous carbon film was initiated by introducing a flow rate of C_2_H_2_ and turning the DC plasma on. At the end of the growth period, the carbon source was turned off and the samples were slowly cooled, within the furnace, under N_2_ gas environment.

### Si microrod array fabrication

For fabricating silicon microrods, a standard planar (1 0 0) Si wafer was patterned by optical lithography. Then, the patterned samples were etched by hydrogen-assisted deep reactive ion etching (DRIE) as reported elsewhere^28^.

### CNT growth

CNTs were grown by DC-PECVD as explained in our previous works^30^.

### Physical and chemical characterization methods

A Hitachi S4160 scanning electron microscope (SEM), a Philips CM300 transmission electron microscope (TEM) and atomic force microscope (AFM) were used to characterize the morphologies as well as micro/nanostructures of the specimens. Selected area electron diffraction (SAED) was also used for examining the crystal structure of *a*-C:N layers. Raman spectra of the deposited *a*-C:N films were obtained by a Bruker Senterra Raman spectrometer equipped with 785 nm LASER diodes. The chemical composition of the *a*-C:N layers was analyzed by X-ray photoelectron spectroscopy (XPS) using an AXIS-ULTRA DLD spectrometer employing an A1 Kα X-ray radiation source at the power of 600 W.

### Electrochemical analysis

The electrochemical performances of the fabricated supercapacitors were evaluated by cyclic voltammetry (CV), galvanostatic charge and discharge (GCD), and Electrochemical Impedance Spectroscopy (EIS) at room temperature. For the impedance measurements a Solarton system was used. All electrochemical measurements were carried out in a standard three electrode cell using a Metrohm Pt counter electrode and a Sentek Ag/AgCl reference electrode. Also, a 0.5 M KCl aqueous solution was used as the electrolyte solution.

## Additional Information

**How to cite this article:** Tali, S. A. S. *et al*. Nitrogen-doped amorphous carbon-silicon core-shell structures for high-power supercapacitor electrodes. *Sci. Rep.* **7**, 42425; doi: 10.1038/srep42425 (2017).

**Publisher's note:** Springer Nature remains neutral with regard to jurisdictional claims in published maps and institutional affiliations.

## Figures and Tables

**Table 1:** The equivalent circuit parameters of the modified sample.

*Rs*	*2* *Ω*
*Rp*	*10* *kΩ*
*T*_*CPE*_	*0.0135*
*n*	*0.9*
*R*_*w*_	*7*
*T*_*w*_	*0.0052*
*p*	*0.45*

## References

[b1] Tiainen V.-M. Amorphous carbon as a bio-mechanical coating-mechanical properties and biological applications. Diam. Relat. Mater. 10, 153–160 (2001).

[b2] Roy R. K. & Lee K.-R. Biomedical applications of diamond-like carbon coatings: A review. J. Biomed. Mater. Res. Part B Appl. Biomater. 83, 72–84 (2007). 17285609 10.1002/jbm.b.30768

[b3] Ferrari A. C. & Robertson J. Raman spectroscopy of amorphous, nanostructured, diamond–like carbon, and nanodiamond. Philos. Trans. R. Soc. London A Math. Phys. Eng. Sci. 362, 2477–2512 (2004). 10.1098/rsta.2004.1452 15482988

[b4] Omer A. M. M. . Photovoltaic characteristics of nitrogen-doped amorphous carbon thin-films grown on quartz and flexible plastic substrates by microwave surface wave plasma CVD. Diam. Relat. Mater. 14, 1084–1088 (2005).

[b5] Liu D. G., Tu J. P., Chen R. & Gu C. D. Microstructure, corrosion resistance and biocompatibility of titanium incorporated amorphous carbon nitride films. Surf. Coatings Technol. 206, 165–171 (2011).

[b6] Ma Z. Q. & Liu B. X. Boron-doped diamond-like amorphous carbon as photovoltaic films in solar cell. Sol. energy Mater. Sol. cells 69, 339–344 (2001).

[b7] Veerasamy V. S., Luten H. A., Petrmichl R. H. & Thomsen S. V. Diamond-like amorphous carbon coatings for large areas of glass. Thin Solid Films 442, 1–10 (2003).

[b8] Hayashi Y., Ishikawa S., Soga T., Umeno M. & Jimbo T. Photovoltaic characteristics of boron-doped hydrogenated amorphous carbon on n-Si substrate prepared by rf plasma-enhanced CVD using trimethylboron. Diam. Relat. Mater. 12, 687–690 (2003).

[b9] Pandolfo A. G. & Hollenkamp A. F. Carbon properties and their role in supercapacitors. J. Power Sources 157, 11–27 (2006).

[b10] Huang Y., Liang J. & Chen Y. An overview of the applications of graphene-based materials in supercapacitors. Small 8, 1805–1834 (2012). 22514114 10.1002/smll.201102635

[b11] Rusop M., Mominuzzaman S. M., Soga T., Jimbo T. & Umeno M. Characterization of phosphorus-doped amorphous carbon and construction of n-carbon/p-silicon heterojunction solar cells. Jpn. J. Appl. Phys. 42, 2339 (2003).

[b12] Chen C. W. & Robertson J. Doping mechanism in tetrahedral amorphous carbon. Carbon N. Y. 37, 839–842 (1999).

[b13] Khun N. W. & Liu E. Effect of substrate temperature on corrosion performance of nitrogen doped amorphous carbon thin films in NaCl solution. Thin Solid Films 517, 4762–4766 (2009).

[b14] Aryal H. R. . Some aspects of nitrogen doped amorphous carbon thin films. in *Photovoltaic Specialists Conference, 2008. PVSC’08. 33rd IEEE* 1–4 (2008).

[b15] Veerasamy V. S. . Photoresponse characteristics of n-type tetrahedral amorphous carbon/p-type Si heterojunction diodes. Appl. Phys. Lett. 64, 2297–2299 (1994).

[b16] Rusop M., Mominuzzaman S. M., Soga T., Jimbo T. & Umeno M. Nitrogen doped n-type amorphous carbon films obtained by pulsed laser deposition with a natural camphor source target for solar cell applications. J. Phys. Condens. Matter 17, 1929 (2005).

[b17] Amaratunga G. A. J. & Silva S. R. P. Nitrogen containing hydrogenated amorphous carbon for thin-film field emission cathodes. Appl. Phys. Lett. 68, 2529–2531 (1996).

[b18] Srinivasan S., Tang Y., Li Y. S., Yang Q. & Hirose A. Ion beam deposition of DLC and nitrogen doped DLC thin films for enhanced haemocompatibility on PTFE. Appl. Surf. Sci. 258, 8094–8099 (2012).

[b19] Park Y. S., Myung H. S., Han J. G. & Hong B. The electrical and structural properties of the hydrogenated amorphous carbon films grown by close field unbalanced magnetron sputtering. Thin Solid Films 482, 275–279 (2005).

[b20] Panwar O. S., Khan M. A., Satyanarayana B. S., Kumar S. & others. Properties of boron and phosphorous incorporated tetrahedral amorphous carbon films grown using filtered cathodic vacuum arc process. Appl. Surf. Sci. 256, 4383–4390 (2010).

[b21] Kocourek T. . DLC coating of textile blood vessels using PLD. Appl. Phys. A 93, 627–632 (2008).

[b22] Bhaviripudi S. . CVD synthesis of single-walled carbon nanotubes from gold nanoparticle catalysts. J. Am. Chem. Soc. 129, 1516–1517 (2007). 17283991 10.1021/ja0673332

[b23] Yu Q. . Graphene segregated on Ni surfaces and transferred to insulators. Appl. Phys. Lett. 93, 113103 (2008).

[b24] Zhu D. . Controllable synthesis of large-area free-standing amorphous carbon films and their potential application in supercapacitors. RSC Adv. 4, 63734–63740 (2014).

[b25] Feng D. . Free-standing mesoporous carbon thin films with highly ordered pore architectures for nanodevices. J. Am. Chem. Soc. 133, 15148–15156 (2011). 21854032 10.1021/ja2056227

[b26] Robertson J. Diamond-like amorphous carbon. Mater. Sci. Eng. R Reports 37, 129–281 (2002).

[b27] Ferrari A. C. & Robertson J. Interpretation of Raman spectra of disordered and amorphous carbon. Phys. Rev. B 61, 14095 (2000).

[b28] Soleimani-Amiri S., Gholizadeh A., Rajabali S., Sanaee Z. & Mohajerzadeh S. Formation of Si nanorods and hollow nano-structures using high precision plasma-treated nanosphere lithography. RSC Adv. 4, 12701–12709 (2014).

[b29] Soleimani-Amiri S. . 3D micro-and nano-machining of hydrogenated amorphous silicon films on SiO_2_/Si and glass substrates. J. Micromechanics Microengineering 25, 74004 (2015).

[b30] Abdi Y., Mohajerzadeh S., Koohshorkhi J., Robertson M. D. & Andrei C. M. A plasma enhanced chemical vapor deposition process to achieve branched carbon nanotubes. Carbon N. Y. 46, 1611–1614 (2008).

[b31] Loh G. C. & Baillargeat D. Graphitization of amorphous carbon and its transformation pathways. J. Appl. Phys. 114, 33534 (2013).

[b32] Dudina D. V. . Nickel-Graphite Composites of Variable Architecture by Graphitization-Accompanied Spark Plasma Sintering and Hot Pressing and their Response to Phase Separation. Sci. Sinter. 47, 237 (2015).

[b33] Conway B. E. Electrochemical supercapacitors: scientific fundamentals and technological applications. (Springer Science & Business Media, 2013).

[b34] Garcia B. B. . Effect of pore morphology on the electrochemical properties of electric double layer carbon cryogel supercapacitors. J. Appl. Phys. 104, 14305 (2008).

[b35] Pell W. G. & Conway B. E. Quantitative modeling of fctors determining Ragone plots for batteries andelectrochemical capacitors. J. Power Sources 63, 255–266 (1996).

[b36] Lee J. A. . Ultrafast charge and discharge biscrolled yarn supercapacitors for textiles and microdevices. Nat. Commun. 4 (2013). 10.1038/ncomms2970 23733169

[b37] Pech D. . Ultrahigh-power micrometre-sized supercapacitors based on onion-like carbon. Nat. Nanotechnol. 5, 651–654 (2010). 20711179 10.1038/nnano.2010.162

[b38] Oakes L. . Surface engineered porous silicon for stable, high performance electrochemical supercapacitors. Sci. Rep. 3 (2013). 10.1038/srep03020 PMC3804850 24145684

[b39] Wu Z.-S., Parvez K., Feng X. & Müllen K. Graphene-based in-plane micro-supercapacitors with high power and energy densities. Nat. Commun. 4 (2013). 10.1038/ncomms3487 PMC3778542 24042088

[b40] Gu W. & Yushin G. Review of nanostructured carbon materials for electrochemical capacitor applications: advantages and limitations of activated carbon, carbide-derived carbon, zeolite-templated carbon, carbon aerogels, carbon nanotubes, onion-like carbon, and graphene. Wiley Interdiscip. Rev. Energy Environ. 3, 424–473 (2014).

[b41] Pikul J. H., Zhang H. G., Cho J., Braun P. V. & King W. P. High-power lithium ion microbatteries from interdigitated three-dimensional bicontinuous nanoporous electrodes. Nat. Commun. 4, 1732 (2013). 23591899 10.1038/ncomms2747

[b42] El-Kady M. F., Strong V., Dubin S. & Kaner R. B. Laser scribing of high-performance and flexible graphene-based electrochemical capacitors. Science (80-). 335, 1326–1330 (2012). 10.1126/science.1216744 22422977

